# Quantitative Parameters of Diffusion Spectrum Imaging: HER2 Status Prediction in Patients With Breast Cancer

**DOI:** 10.3389/fonc.2022.817070

**Published:** 2022-02-03

**Authors:** Chunping Mao, Wei Jiang, Jiayi Huang, Mengzhu Wang, Xu Yan, Zehong Yang, Dongye Wang, Xiang Zhang, Jun Shen

**Affiliations:** ^1^ Department of Radiology, Sun Yat-Sen Memorial Hospital, Sun Yat-Sen University, Guangzhou, China; ^2^ Department of Radiology, Shenshan Central Hospital, Sun Yat-Sen Memorial Hospital, Sun Yat-Sen University, Shanwei, China; ^3^ MR Scientific Marketing, Siemens Healthcare, Guangzhou, China

**Keywords:** breast cancer, diffusion magnetic resonance imaging, diffusion spectrum imaging, HER2 status, receiver operating characteristic

## Abstract

**Objective:**

To explore the value of quantitative parameters derived from diffusion spectrum imaging (DSI) in preoperatively predicting human epidermal growth factor receptor 2 (HER2) status in patients with breast cancer.

**Methods:**

In this prospective study, 114 and 56 female patients with invasive ductal carcinoma were consecutively included in a derivation cohort and an independent validation cohort, respectively. Each patient was categorized into HER2-positive or HER2-negative groups based on the pathologic result. All patients underwent DSI and conventional MRI including dynamic contrast-enhanced MRI (DCE-MRI) and diffusion-weighted imaging (DWI). The tumor size, type of the time-signal intensity curve (TIC) from DCE-MRI, apparent diffusion coefficient (ADC) from DWI, and quantitative parameters derived from DSI, including diffusion tensor imaging (DTI), diffusion kurtosis imaging (DKI), mean apparent propagator (MAP), and neurite orientation dispersion and density imaging (NODDI) of primary tumors, were measured and compared between the HER2-positive and HER2-negative groups in the derivation cohort. Univariable and multivariable logistic regression analyses were used to determine the potential independent predictors of HER2 status. The discriminative ability of quantitative parameters was assessed by receiver operating characteristic (ROC) curve analyses and validated in the independent cohort.

**Results:**

In the derivation cohort, the tumor size, TIC type, and ADC values did not differ between the HER2-positive and HER2-negative groups (*p* = 0.126–0.961). DSI quantitative parameters including axial kurtosis of DKI (DKI_AK), non-Gaussianity (MAP_NG), axial non-Gaussianity (MAP_NG_Ax_), radial non-Gaussianity (MAP_NG_Rad_), return-to-origin probability (MAP_RTOP), return-to-axis probability of MAP (MAP_RTAP), and intracellular volume fraction of NODDI (NODDI_ICVF) were lower in the HER2-positive group than in the HER2-negative group (*p* ≤ 0.001–0.035). DSI quantitative parameters including radial diffusivity (DTI_RD), mean diffusivity of DTI (DTI_MD), mean squared diffusion (MAP_MSD), and q-space inverse variance of MAP (MAP_QIV) were higher in the HER2-positive group than in the HER2-negative group (*p* = 0.016–0.049). The ROC analysis showed that the area under the curve (AUC) of ADC was 0.632 and 0.568, respectively, in the derivation and validation cohorts. The AUC values of DSI quantitative parameters ranged from 0.628 to 0.700 and from 0.673 to 0.721, respectively, in the derivation and validation cohorts. Logistic regression analysis showed that only NODDI_ICVF was an independent predictor of HER2 status (*p* = 0.001), with an AUC of 0.700 and 0.721, respectively, in the derivation and validation cohorts.

**Conclusions:**

DSI could be helpful for preoperative prediction of HER2, but DSI alone may not be sufficient in predicting HER2 status preoperatively in patients with breast cancer.

## Highlights

• DSI quantitative parameters may be useful for preoperative prediction of the HER2 status in patients with breast cancer.
• DSI quantitative parameters have a better discriminative ability to predict the HER2 status than the ADC value derived from DWI.
• DSI quantitative parameters alone may not be sufficient in predicting the HER2 status in patients with breast cancer.

## Introduction

Breast cancer is the most common malignancy in women worldwide, which accounts for approximately 11.6% of all malignancies with an increasing trend (1). It is a highly heterogeneous malignancy with a variety of biological characteristics. In recent years, the therapeutic paradigm of breast cancer is changing from conventional therapy to personalized treatment, which is based on its molecular subtypes (2). Human epidermal growth factor receptor 2 (HER2) is a transmembrane tyrosine kinase receptor with a pathologic characteristic of promoting tumor angiogenesis and enhancing tumor invasiveness, and it generally shows high expression and gene amplification in malignant epithelial tumors of the breast (3). As a major classifier of molecular subtypes, HER2 is the therapeutic target of breast cancer with a positive rate of 15%–30% (4). In addition, HER2-positive status was an independent risk factor for the prognosis of patients with breast cancer (5). Patients with early HER2-positive breast cancer had significantly low risks of recurrence and mortality (6). However, HER2 status is usually determined by invasive procedures such as surgery and biopsy. A non-invasive means capable of predicting HER2 status would be of great clinical relevance for breast cancer patients.

Diffusion MRI allows non-invasive mapping of the diffusion process of water molecules in biological tissues *in vivo* (7). Diffusion-weighted imaging (DWI) and diffusion tensor imaging (DTI) are the standard models of the diffusion MRI most commonly used in the clinic. More advanced models such as diffusion kurtosis imaging (DKI), mean apparent propagator (MAP), and neurite orientation dispersion and density imaging (NODDI) can be derived from diffusion spectrum imaging (DSI). DSI is a model freely reconstructed by using multiple b-values and gradient directions in the entire q-space to sample diffusion signals of water molecules and to quantitatively estimate them by probability density function (8), which is mathematically and physically superior to other diffusion MRI techniques (9). Clinically, it has been used in the diseases of the central nervous system (10, 11). In breast cancer, some diffusion models, including DWI, DTI, and DKI, have been applied to predict the HER2 status but showing a poor performance (12–15), partly due to the limitation of specific model assumptions (9). MAP and NODDI models are characterized by describing more complex microstructures (7, 16), which can provide more informative quantitative metrics, such as MAP-based parameters non-Gaussianity (MAP_NG) and NODDI-based parameters intracellular volume fraction (NODDI_ICVF). However, whether quantitative parameters derived from DSI can be used to preoperatively predict HER2 status remains unknown.

In this preliminary, prospective study, quantitative parameters derived from DSI of primary tumors in female patients with breast cancer were analyzed. The purpose of our study was to explore the predictive value of quantitative DSI parameters in preoperatively predicting HER2 status in patients with breast cancer.

## Materials and Methods

### Study Participants

The institutional review board of the Sun Yat-Sen Memorial Hospital, Sun Yat-Sen University, approved this study (SYSEC-KY-KS-2021-182), and all participants provided written informed consents before enrollment in this study.

**Figure 1:**
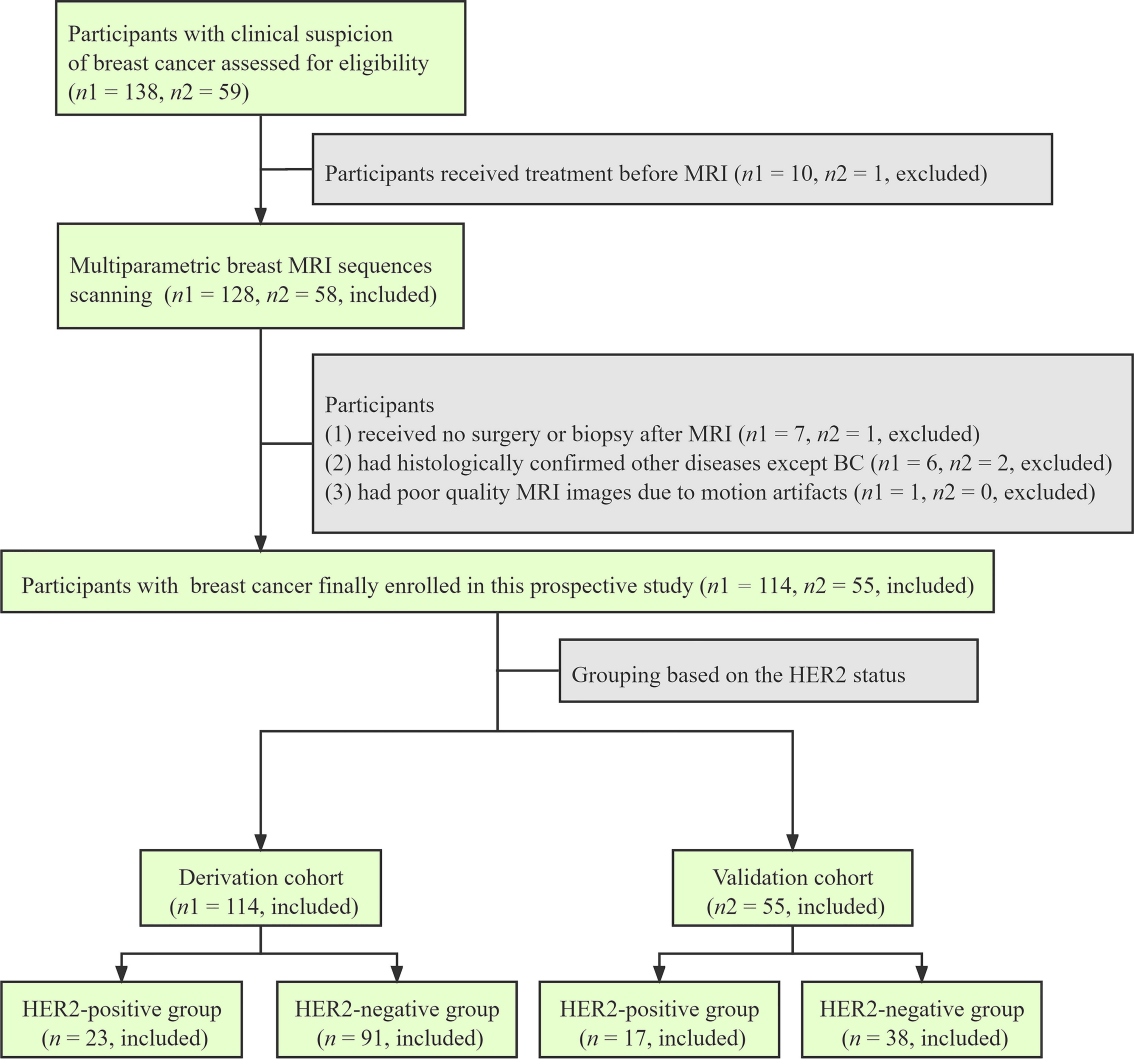
Flowchart shows the enrollment of the participants in the derivation and validation cohorts.

From November 2020 to May 2021, 138 female participants with clinical suspicion of breast cancer were recruited from our hospital. All participants met the following inclusion criteria: 1) pathologic type and HER2 status in breast cancer were identified by surgical or biopsy pathology and 2) no previous treatment before MRI. The exclusion criteria were as follows: 1) patients had claustrophobia and were allergic to the contrast agents and 2) poor quality of MRI images with motion artifacts. Twenty-four participants were excluded, and finally, 114 female participants with breast cancer were included as a derivation cohort. From May 2021 to October 2021, another 56 participants were enrolled into an independent validation cohort from our hospital based on the above inclusion and exclusion criteria. According to histologic results, each participant was categorized into the HER2-positive group or the HER2-negative group. The flowchart of participants’ enrollment is shown in **Figure 1**.

### MRI Protocol

All participants underwent breast multiparametric MRI within 1 week before surgery on a 3.0-T MR scanner (MAGNETOM Skyra; Siemens Healthcare, Erlangen, Germany) equipped with a dedicated breast coil. The imaging sequences included axial three-dimensional volumetric interpolated breath-hold examination (VIBE) T1-weighted imaging (T1WI), axial and sagittal turbo spin-echo with short tau inversion recovery T2-weighted imaging (T2WI), axial DWI (b = 0, 800 s/mm^2^) with spectral attenuated inversion recovery (SPAIR) fat saturation, axial DSI, and axial dynamic contrast-enhanced MRI (DCE-MRI). DSI was acquired before DCE-MRI using a spin-echo echo-planar imaging (SE-EPI) sequence with SPAIR for fat saturation. Totally, 9 b-values (0, 200, 450, 650, 900, 1,100, 1,350, 1,800, and 2,000 s/mm^2^) were applied along with 2, 6, 12, 8, 6, 24, 24, 12, and 6 directions, respectively. DCE-MRI consisted of 40 dynamic phases of measurements with a temporal resolution of 8 s was performed using an acceleration-VIBE (CAIPIRINHA-VIBE) sequence. At the end of the second phase of dynamic acquisition, gadodiamide (Gd-DTPA-BMA; Omniscan, GE Healthcare, Chicago, IL, USA) was intravenously administrated at a dose of 0.1 mmol/kg and a flow rate of 3.5 ml/s. The detailed acquisition parameters of multiparametric MRI sequences are shown in **Table 1**.

**Table 1:** Multiparametric MRI sequences and acquisition parameters.

Sequence	TR (ms)	TE (ms)	FOV (mm)	Matrix	Slice thickness (mm)	Slice gap (mm)	Fat suppression	Flip angle	b value (s/mm^2^)	Acquisition time
T1WI	5.38	2.46/3.69	380 × 380	384 × 384	1.5	0	Yes	8°	–	1 min 59 s
T2WI	7,600	75	340 × 340	358 × 448	4	0.8	Yes	116°	–	3 min 27 s
DWI	7,620	64/104	360 × 293	156 × 192	4	0.8	Yes	180°	0/800	1 min 54 s
DCE-MRI	3.25	1.22	380 × 326	187 × 256	2.5	1.5	Yes	11°	–	7 min 10 s
DSI	6,600	97	350 × 350	174 × 174	4	0.8	Yes	90°	0–2,000	11 min 33 s

b value = 0–2,000, including 0, 200, 450, 650, 900, 1,100, 1,350, 1,800, and 2,000 s/mm^2^.

T1WI, T1-weighted imaging; T2WI, T2-weighted imaging; DWI, diffusion-weighted imaging; DCE-MRI, dynamic contrast-enhanced MRI; DSI, diffusion spectrum imaging; TR, repetition time; TE, echo time; FOV, field of view.

### Image Analysis

**Figure 2:**
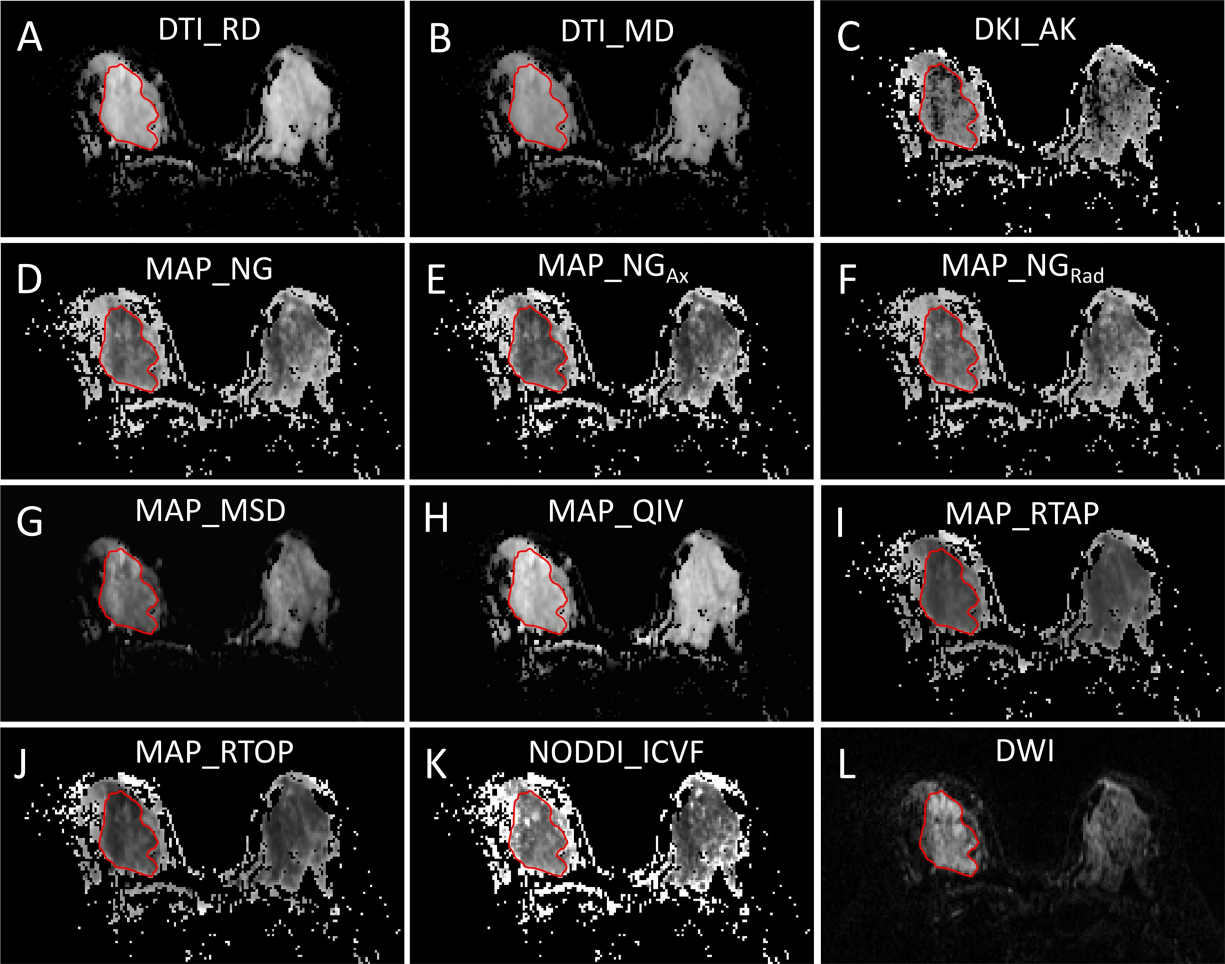
A 44-year-old woman with pathologically confirmed HER2-negative breast cancer in the right breast. **(A–K)** Quantitative DSI parameter measurements and **(L)** diffusion-weighted imaging (DWI) measurement.

**Figure 3:**
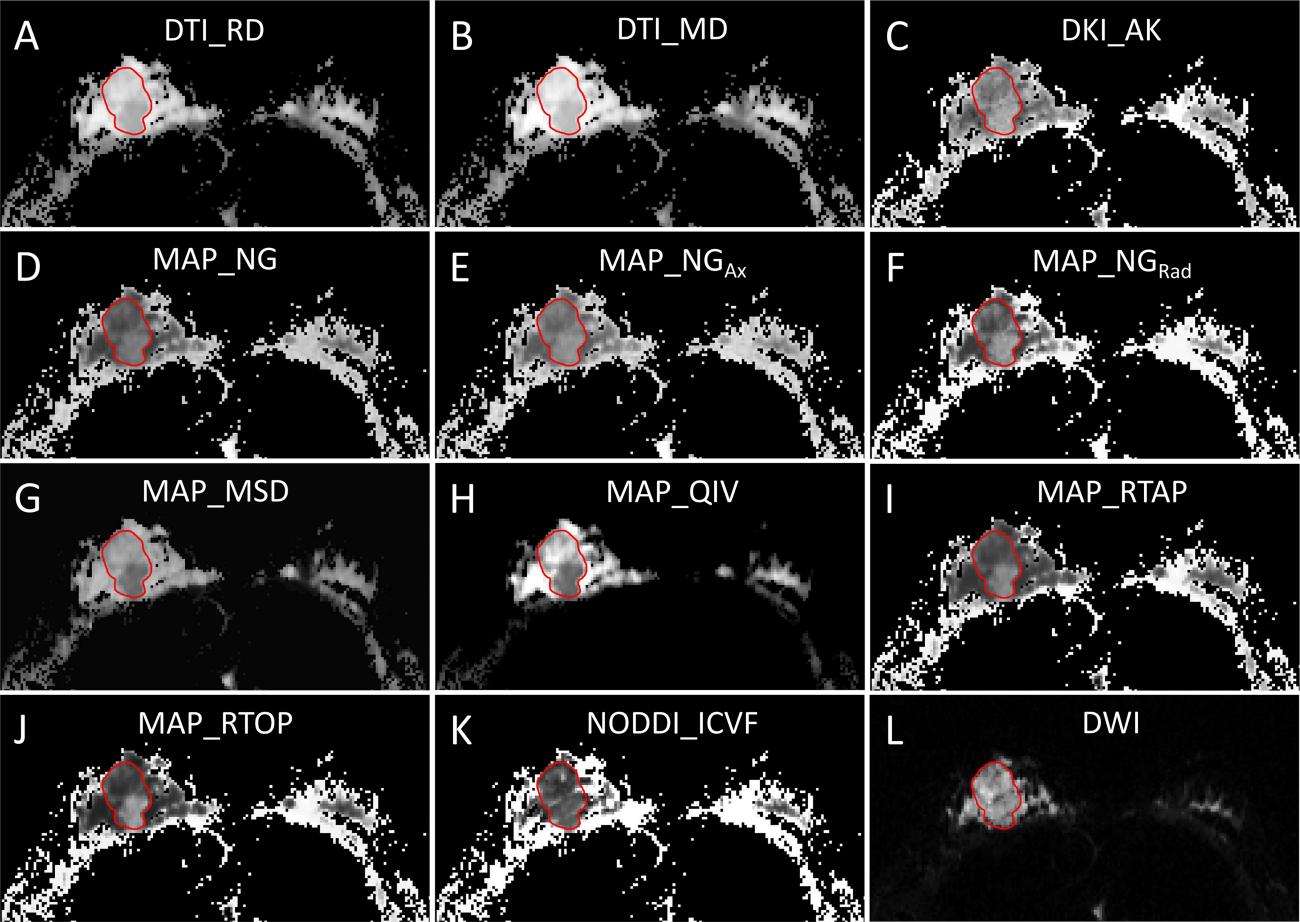
A 42-year-old woman with pathologically confirmed human epidermal growth factor receptor 2 (HER2)-positive breast cancer in the right breast. **(A–K)** Diffusion spectrum imaging (DSI) quantitative parameter measurements and **(L)** diffusion-weighted imaging (DWI) measurement.

DSI quantitative parameters of primary tumors, including four DTI-based parameters [fractional anisotropy (DTI_FA), axial diffusivity (DTI_AD), radial diffusivity (DTI_RD), and mean diffusivity (DTI_MD)], seven DKI-based parameters [DKI_FA, DKI_AD, DKI_RD, DKI_MD, axial kurtosis (DKI_AK), radial kurtosis (DKI_RK), and mean kurtosis (DKI_MK)], eight MAP-based parameters [non-Gaussianity (MAP_NG), axial non-Gaussianity (MAP_NG_Ax_), radial non-Gaussianity (MAP_NG_Rad_), mean squared diffusion (MAP_MSD), q-space inverse variance (MAP_QIV), return-to-origin probability (MAP_RTOP), return-to-axis probability (MAP_RTAP), and return-to-plane probability (MAP_RTPP)], and three NODDI-based parameters [intracellular volume fraction (NODDI_ICVF), volume fraction of the isotropic compartment (NODDI_ISOVF), and orientation dispersion index (NODDI_ODI)] were calculated from DSI data using an in-house developed software NeuDiLab, which is rooted in an open-source tool DIPY (Diffusion Imaging In Python, https://dipy.org/). To measure quantitative parameters, regions of interest (ROIs) were delineated by two radiologists (XZ and JH, who had 8 years and 2 years of experience in diagnostic breast imaging, respectively) using the ITK-SNAP software (http://www.itksnap.org/pmwiki/pmwiki.php) (17). The ROIs were drawn along the edge of the tumor on DCE images acquired at 88 s after the injection of contrast agent and on the section showing the maximal dimension of the primary tumor and then automatically copied to images of the quantitative DSI parameters (**Figures 2** and **3**). Both radiologists knew the diagnosis of breast cancer but were blinded to the pathologic HER2 status. For each participant, the apparent diffusion coefficient (ADC) value of the primary tumor was also calculated, and the ROIs of which were delineated by copying the ROIs of DSI as templates. In addition, the tumor size (maximal diameter) of the primary tumor was measured, and the time-signal intensity curve (TIC) was derived from DCE-MRI by one of the two radiologists (XZ). TIC was classified into three types, including steady curve (type I), plateau curve (type II), and washout curve (type III), as previously described (18).

### Pathologic Evaluation

All breast cancer specimens were processed in accordance with the standards published by the American Society of Clinical Oncology and College of American Pathologists (19). The pathologic characteristics, including HER2, estrogen receptor (ER) and progesterone receptor (PR) statuses, and Ki-67 index were recorded. HER2 status was scored depending on the membranous staining pattern and percentage of stained malignant cells, including four grades, −, +, ++, and +++, in which HER2 − and + were judged as HER2 negative and HER2 +++ was judged as HER2 positive in immunohistochemistry specimens (19). In addition, HER2 ++ was further verified by fluorescence *in situ* hybridization (FISH) with gene amplification defined as HER2 positive and without gene amplification defined as HER2-negative (19). ER and PR statuses were determined by the percent of stained tumor cells with a cutoff for positive higher than 1% (20). Ki-67 index was reported by the percentage of immunoreactive cells with a cutoff of 14% (21).

### Statistical Analysis

The normality of data distribution was determined by the Shapiro–Wilk test, with variance equality checked by Levene’s test. Continuous variables are expressed as means ± SD. Intraclass correlation coefficient (ICC) was calculated to assess the inter-observer reliability in the measurement of quantitative parameters. Values of ICC greater than 0.9, between 0.75 and 0.9, between 0.5 and 0.75, and less than 0.5 indicated excellent, good, moderate, and poor agreement, respectively. Quantitative DSI parameters, tumor size, and TIC types between the HER2-positive and HER2-negative groups were compared by independent *t*-tests, *chi-square* test, or Mann–Whitney *U* test. Univariable and multivariable logistic analyses were used to determine independent predictors of HER2 status from DSI parameters, tumor size, and TIC type. The receiver operating characteristic (ROC) curves were used to assess the discriminative ability of significant parameters for HER2 status, and the area under the curve (AUC) values were obtained and compared using the DeLong test. The cutoff value, sensitivity, specificity, and accuracy were calculated and expressed by using their two-sided 95% CIs. Statistical significance was set as a two-sided *p*-value of less than 0.05. Statistical analyses were analyzed with SPSS, version 26.0 (SPSS Inc., Chicago, IL, USA).

## Results

### Demographic and Clinical Characteristics of Participants

The demographic and clinical characteristics of participants in the derivation and validation cohorts are shown in **Table 2**. Among 114 patients in the derivation cohort, 23 patients had a HER2-positive invasive ductal carcinoma, and 91 patients had a HER2-negative invasive ductal carcinoma. ER and PR statuses were significantly different between these two groups (*p* = 0.012 and 0.001, respectively). There were no statistically significant differences in age, menarche, menopause, childbearing, family history, and Ki-67 between the two groups (*p* = 0.12–0.799). Among 55 patients in the validation cohort, 17 patients had a HER2-positive invasive ductal carcinoma, and 38 patients had a HER2-negative invasive ductal carcinoma. ER and PR statuses were also significantly different between these two groups (*p* = 0.003 and 0.012, respectively). There were no statistically significant differences in age, menarche, menopause, childbearing, family history, and Ki-67 between the two groups (*p* = 0.145–0.500).

**Table 2:** Demographic and clinical characteristics of participants in the derivation and validation cohorts.

Characteristics		Derivation cohort		*p*-Value	Validation cohort		*p*-Value
		HER-2 positive group (n = 23)	HER-2 negative group (n = 91)		HER-2 positive group (n = 17)	HER-2 negative group (n = 38)	
Age (years)		48.13 ± 11.19	48.96 ± 10.65	0.743^a^	51.06 ± 11.58	46.71 ± 11.87	0.211^a^
Menstrual History	Menarche (years)	13.74 ± 1.82	13.65 ± 1.45	0.799^a^	13.00 ± 0.00	13.32 ± 1.00	0.145^b^
	Menopause (yes/no)	4/19	31/60	0.121^c^	9/8	15/23	0.352^c^
Childbearing (yes/no)		22/1	83/8	0.480^c^	17/0	36/2	0.335^c^
Family history of breast cancer (yes/no)		3/20	9/82	0.660^c^	0/17	2/36	0.335^c^
ER status (positive/negative)		11/12	68/23	0.012^c*^	6/11	29/9	0.003^c*^
PR status (positive/negative)		6/17	58/33	0.001^c*^	6/11	27/11	0.012^c*^
Ki-67 index (>14%/≤14%)		21/2	80/11	0.647^c^	17/0	37/1	0.500^c^

The continuous variables are expressed as mean ± SD.

ER, estrogen receptor; PR, progesterone receptor.

a The p-value was obtained by the t-test.

b The p-value was obtained by the Mann–Whitney U test.

c The p-value was obtained by the chi-square test.

^*^p < 0.05 was considered statistically significant.

### Comparisons of Conventional MRI and Quantitative Diffusion Spectrum Imaging Parameters

**Figure 4:**
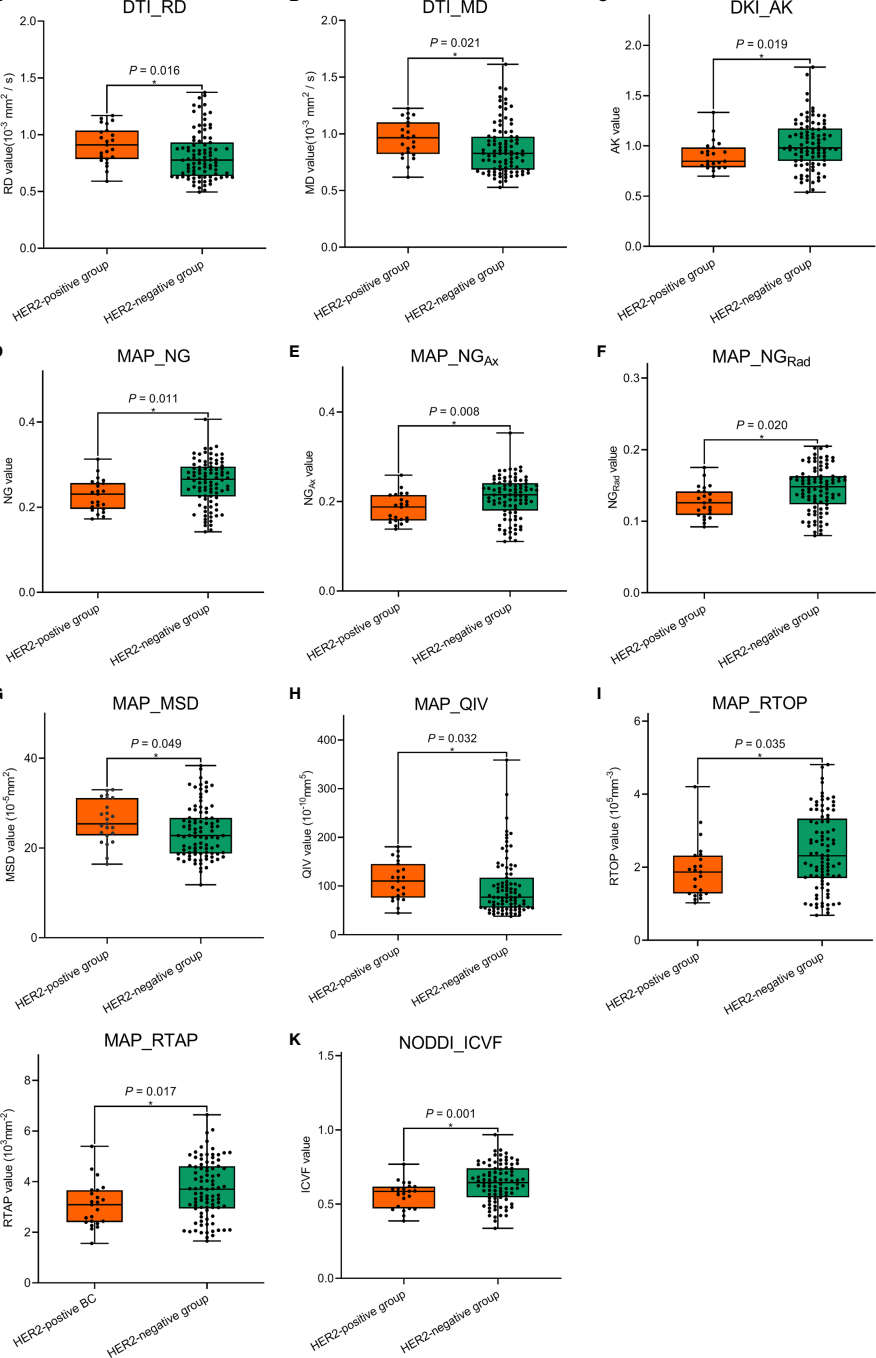
Comparisons of the significant diffusion spectrum imaging (DSI) quantitative parameters **(A–K)** between the human epidermal growth factor receptor 2 (HER2)-positive group and the HER2-negative group in the derivation cohort. * The statistically significant level, *p* < 0.05.

**Figure 5:**
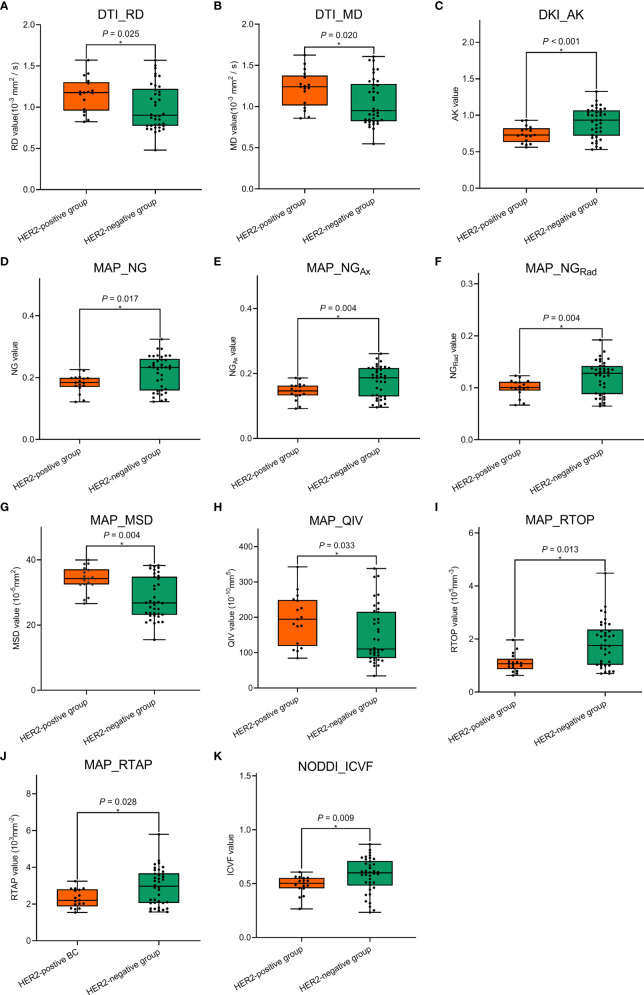
Comparisons of the significant diffusion spectrum imaging (DSI) quantitative parameters **(A–K)** between the human epidermal growth factor receptor 2 (HER2)-positive group and the HER2-negative group in the validation cohort. * The statistically significant level, *p* < 0.05.

The ICCs of ADC value and DSI quantitative parameters measurement by two radiologists ranged from 0.908 to 0.990, indicating excellent agreements. Therefore, the average values of these parameters measured by two radiologists were finally calculated for the subsequent analyses. Comparisons of the tumor size, TIC type, ADC value, and DSI quantitative parameters between the HER2-positive and HER2-negative groups are shown in **Table 3**, **Figures 4**, and **5**. In the derivation cohort, ADC values did not statistically differ between these two groups (*p* = 0.126). The DKI_AK, MAP_NG, MAP_NG_Ax_, MAP_NG_Rad_, MAP_RTOP, MAP_RTAP, and NODDI_ICVF values were lower in the HER2-positive group than in the HER2-negative group (*p* = 0.001–0.035), while the DTI_MD, DTI_RD, MAP_MSD, and MAP_QIV values were higher in the HER2-positive group than in HER2-negative group (*p* = 0.016–0.049). No statistically significant difference was found in other DSI quantitative parameters between the two groups (*p* = 0.054–0.874). Similar results were found in the validation cohort.

**Table 3:** Comparisons of conventional MRI and quantitative DSI parameters between HER2-positive and HER2-negative groups.

Parameters		Derivation cohort		*p*-Value	Validation cohort		*p*-Value
		HER2-positive (n = 23)	HER2-negative (n = 91)		HER2-positive (n = 17)	HER2-negative (n = 38)	
**DCE-MRI**							
Tumor size	Diameter (mm)	32.60 ± 16.82	30.21 ± 15.75	0.474^b^	32.60 ± 16.82	30.21 ± 15.75	0.474^b^
	>4 mm/<4 mm	17/6	18/76	0.507^c^	6/11	10/28	0.459^c^
TIC (type II/type III)		12/11	48/43	0.961^c^	12/4	20/18	0.095^c^
**DWI**							
ADC (10^−3^ mm^2/^s)		982.98 ± 233.20	1,058.43 ± 263.57	0.126^b^	1,035.48 ± 227.04	1,079.29 ± 204.46	0.423^b^
**DSI**							
DTI_FA		0.10 ± 0.03	0.12 ± 0.06	0.172^b^	0.10 ± 0.03	0.10 ± 0.04	0.813^b^
DTI_AD (10^−3^ mm^2/^s)		1.04 ± 0.17	0.97 ± 0.25	0.054^b^	1.35 ± 0.25	1.16 ± 0.28	0.023^b*^
DTI_RD (10^−3^ mm^2/^s)		0.91 ± 0.16	0.82 ± 0.21	0.016^b*^	1.15 ± 0.21	1.00 ± 0.27	0.025^b*^
DTI_MD (10^−3^ mm^2/^s)		1.00 ± 0.16	0.87 ± 0.22	0.021^b*^	1.21 ± 0.22	1.05 ± 0.28	0.020^b*^
DKI_FA		0.14 ± 0.04	0.16 ± 0.07	0.172^b^	0.14 ± 0.04	0.14 ± 0.05	0.863^b^
DKI_AD (10^−3^ mm^2/^s)		1.55 ± 0.28	1.46 ± 0.36	0.125^b^	1.99 ± 0.36	1.73 ± 0.37	0.021^b*^
DKI_RD (10^−3^ mm^2/^s)		1.28 ± 0.24	1.17 ± 0.30	0.059^b^	1.62 ± 0.27	1.43 ± 0.36	0.021^b*^
DKI_MD (10^−3^ mm^2/^s)		1.37 ± 0.25	1.27 ± 0.32	0.083^b^	1.74 ± 0.30	1.53 ± 0.36	0.056^b^
DKI_AK		0.90 ± 0.14	1.01 ± 0.24	0.019^b*^	0.79 ± 0.10	1.06 ± 0.15	0.000^a*^
DKI_RK		0.70 ± 0.12	0.77 ± 0.21	0.166^a^	0.64 ± 0.11	0.69 ± 0.18	0.167^a^
DKI_MK		0.78 ± 0.10	0.86 ± 0.21	0.064^b^	0.68 ± 0.11	0.77 ± 0.18	0.084^a^
MAP_NG		0.23 ± 0.04	0.26 ± 0.05	0.011^a*^	0.18 ± 0.03	0.22 ± 0.06	0.017^b*^
MAP_NG_Ax_		0.18 ± 0.03	0.21 ± 0.05	0.008^b*^	0.15 ± 0.03	0.18 ± 0.05	0.004^a*^
MAP_NG_Rad_		0.13 ± 0.2	0.15 ± 0.03	0.020^a*^	0.10 ± 0.02	0.12 ± 0.03	0.004^a*^
MAP_MSD (10^−5^ mm^2^)		25.91 ± 4.73	23.64 ± 5.86	0.049^b*^	34.04 ± 3.89	28.40 ± 6.45	0.004^b*^
MAP_QIV (10^−10^ mm^5^)		110.55 ± 39.20	97.09 ± 58.49	0.032^b*^	193.46 ± 71.41	150.47 ± 80.37	0.033^b*^
MAP_RTOP (10^5^ mm^−3^)		1.91 ± 0.77	2.44 ± 1.05	0.035^b*^	1.11 ± 0.34	1.78 ± 0.90	0.013^b*^
MAP_RTAP (10^3^ mm^−2^)		3.08 ± 0.89	3.70 ± 1.14	0.017^a*^	2.31 ± 0.50	2.93 ± 1.01	0.028^b*^
MAP_RTPP (10^1^ mm^−1^)		4.82 ± 0.58	5.11 ± 0.77	0.102^a^	4.15 ± 0.43	4.58 ± 0.69	0.008^a*^
NODDI_ICVF		0.56 ± 0.09	0.64 ± 0.13	0.001^a*^	0.49 ± 0.08	0.58 ± 0.16	0.009^a*^
NODDI_ISOCF		0.37 ± 0.11	0.34 ± 0.13	0.339^a^	0.52 ± 0.11	0.45 ± 0.14	0.080^b^
NODDI_ODI		0.60 ± 0.08	0.60 ± 0.13	0.874^b^	0.47 ± 0.12	0.54 ± 0.12	0.044^a*^

The continuous variables are expressed as mean ± SD.

DCE-MRI, dynamic contrast-enhanced MRI; TIC, time-signal intensity curve; DWI, diffusion-weighted imaging; ADC, apparent diffusion coefficient; DSI, diffusion spectrum imaging; DTI, diffusion tensor imaging; DKI, diffusion kurtosis imaging; MAP, mean apparent propagator; NODDI, neurite orientation dispersion and density imaging; FA, fractional anisotropy; AD, axial diffusivity; RD, radial diffusivity; MD, mean diffusivity; AK, axial kurtosis; RK, radial kurtosis; MK, mean kurtosis; NG, non-Gaussianity; NG_Ax_, axial non-Gaussianity; NG_Rad_, radial non-Gaussianity; MSD, mean squared diffusion; QIV, q-space inverse variance; RTOP, return-to-origin probability; RTAP, return-to-axis probability; RTPP, return-to-plane probability; ICVF, intracellular volume fraction; ISOVF, volume fraction of the isotropic compartment; ODI, orientation dispersion index.

a The p-value was obtained by the t-test.

b The p-value was obtained by the Mann–Whitney U test.

c The p-value was obtained by the chi-square test.

^*^p < 0.05 was considered statistically significant.

### Diagnostic Performances

**Figure 6:**
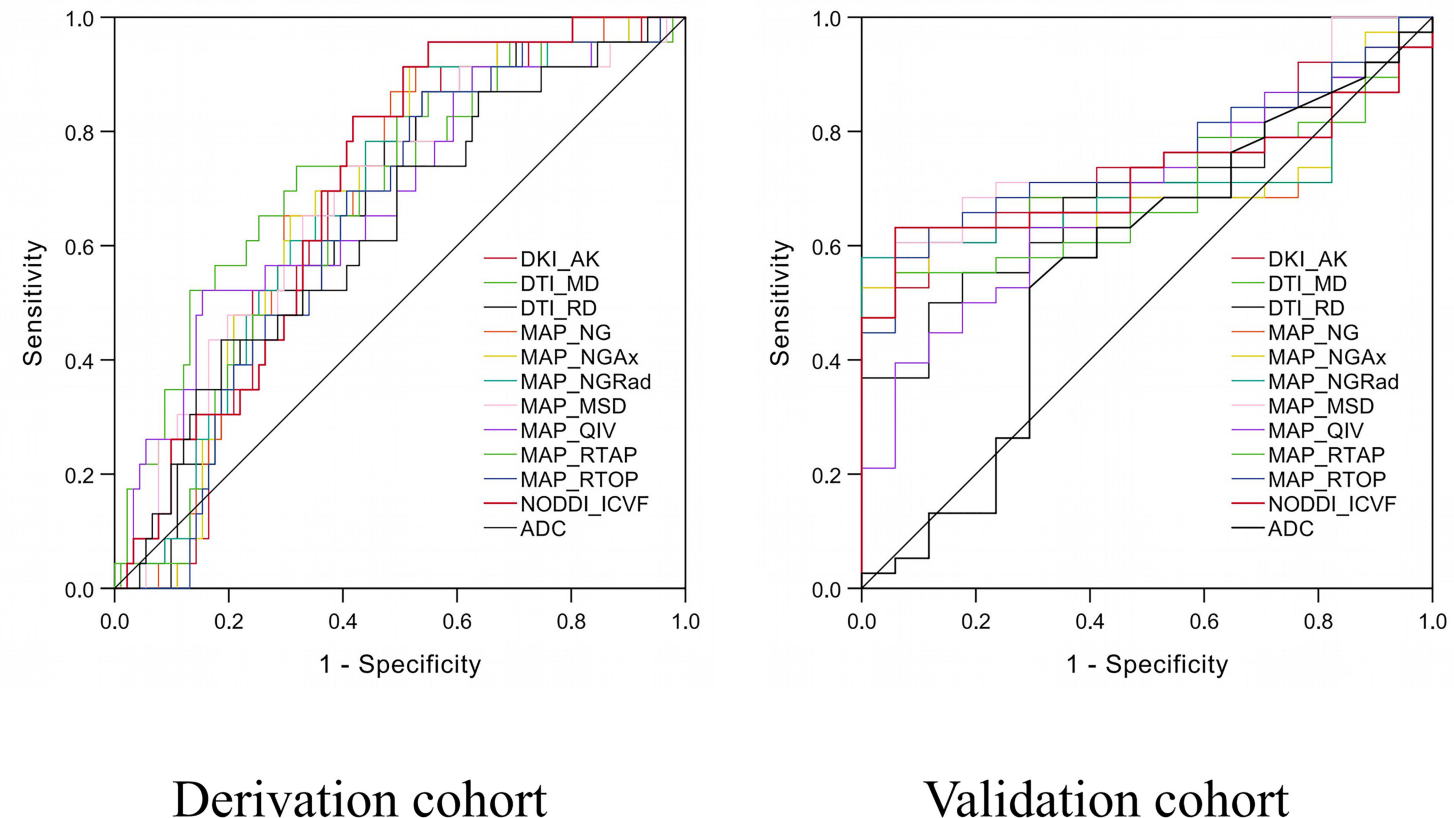
Receiver operating characteristic (ROC) curves of diffusion spectrum imaging (DSI) quantitative parameters and apparent diffusion coefficient (ADC) value for predicting human epidermal growth factor receptor 2 (HER2) status in the derivation and validation cohorts.

ROC analyses of conventional MRI and quantitative DSI parameters in discriminating HER2 status are shown in **Table 4** and **Figure 6**. In the derivation cohort, the AUC of ADC was 0.632. The AUC values of DSI quantitative parameters ranged from 0.628 to 0.700. Among all the DSI quantitative parameters, NODDI_ICVF had the highest AUC of 0.700. There was no statistically significant difference in AUC values between NODDI_ICVF and ADC value (*p* = 0.097). There were statistically significant differences in AUC values between NODDI_ICVF and DKI_AK, DTI_MD, DTI_RD, MAP_NG, MAP_NG_AX_, MAP_NG_RAD_, and MAP_RTAP (*p* = 0.016–0.046) and no statistically significant difference in AUC values between NODDI_ICVF and MAP_MSD, MAP_QIV, and MAP_RTOP (*p* = 0.055–0.067). The ADC and DSI quantitative parameters had a sensitivity between 43.5% and 91.3%, a specificity between 41.8% and 81.3%, and an accuracy between 51.8% and 73.7%. Univariable and multivariable logistic regression analyses (**Table 5**) showed that among all DSI quantitative parameters, tumor size, and TIC type, only NODDI_ICVF was an independent predictor of HER2 status (*p* = 0.001). In the validation cohorts, the AUC values of DTI_RD, DTI_MD, DKI_AK, MAP_NG, MAP_NG_Ax_, MAP_NG_Rad_, MAP_MSD, MAP_QIV, MAP_RTOP, MAP_RTAP, and NODDI_ICVF ranged from 0.673 to 0.721. NODDI_ICVF also had the highest AUC of 0.721.

**Table 4:** ROC analyses of conventional MRI and quantitative DSI parameters in discriminating HER2 status in patients with breast cancer.

Parameters		Derivation cohort					Validation cohort				
		Cutoff value	AUC	Sensitivity (%)	Specificity (%)	Accuracy (%)	Cutoff value	AUC	Sensitivity (%)	Specificity (%)	Accuracy (%)
**DWI**											
ADC (10^−3^ mm^2/^s)		818.955	0.632 (0.536–0.720)	43.5 (0.232–0.655)	81.3 (0.719–0.887)	73.7 (0.645–0.813)	989.900	0.568 (0.428–0.701)	70.6 (0.440–0.897)	57.9 (0.408–0.737)	67.3 (0.532–0.790)
**DSI**											
DTI_MD (10^−3^ mm^2^/s)		0.782	0.656 (0.561–0.742)	91.3 (0.720–0.989)	41.8 (0.315–0.526)	51.8 (0.422–0.611)	1.182	0.698 (0.559–0.815)	70.6 (0.440–0.897)	68.4 (0.513–0.825)	67.3 (0.532–0.790)
DTI_RD (10^−3^ mm^2^/s)		0.750	0.628 (0.532–0.717)	82.6 (0.612–0.950)	53.8 (0.420–0.633)	54.4 (0.448–0.637)	0.898	0.690 (0.551–0.808)	88.2 (0.636–0.985)	50.0 (0.334–0.666)	67.3 (0.532–0.790)
DKI_AK		0.954	0.658 (0.564–0.745)	73.9 (0.516–0.898)	60.4 (0.496–0.705)	63.2 (0.536–0.719)	0.932	0.673 (0.534–0.794)	100.0 (0.805–1.000)	44.7 (0.286–0.617)	63.6 (0.495–0.759)
MAP_NG		0.263	0.686 (0.592–0.770)	87.0 (0.664–0.972)	51.7 (0.409–0.623)	58.8 (0.492–0.678)	0.227	0.703 (0.564–0.818)	100.0 (0.805–1.000)	57.9 (0.408–0.737)	69.1 (0.550–0.805)
MAP_NG_Ax_		0.219	0.679 (0.585–0.763)	91.3 (0.720–0.989)	48.4 (0.377–0.591)	57.0 (0.474–0.661)	0.186	0.701 (0.563–0.817)	100.0 (0.805–1.000)	52.6 (0.358–0.690)	69.1 (0.550–0.805)
MAP_NG_Rad_		0.151	0.684 (0.590–0.768)	91.3 (0.720–0.989)	49.5 (0.388–0.601)	57.9 (0.483–0.670)	0.123	0.707 (0.569–0.822)	100.0 (0.805–1.000)	57.9 (0.408–0.737)	69.1 (0.550–0.805)
MAP_MSD (10^−5^ mm^2^)		20.805	0.633 (0.538–0.721)	91.3 (0.720–0.989)	41.8 (0.315–0.526)	51.8 (0.422–0.611)	27.503	0.690 (0.551–0.808)	82.4 (0.566–0.962)	55.3 (0.383–0.714)	69.1 (0.550–0.805)
MAP_QIV (10^−10^ mm^5^)		69.518	0.645 (0.550–0.733)	91.3 (0.720–0.989)	41.8 (0.315–0.526)	51.8 (0.422–0.611)	170.438	0.681 (0.542–0.800)	70.6 (0.440–0.897)	63.2 (0.460–0.782)	69.1 (0.550–0.805)
MAP_RTOP (10^5^ mm^−3^)		2.445	0.643 (0.547–0.730)	87.0 (0.664–0.972)	46.2 (0.356–0.569)	54.4 (0.448–0.637)	1.643	0.686 (0.546–0.804)	100.0 (0.805–1.000)	44.7 (0.286–0.617)	70.9 (0.569–0.820)
MAP_RTAP (10^3^ mm^−2^)		3.686	0.654 (0.559–0.741)	82.6 (0.612–0.950)	50.6 (0.399–0.612)	57.0 (0.474–0.661)	4.761	0.687 (0.548–0.806)	94.1 (0.713–0.999)	55.3 (0.383–0.714)	69.1 (0.550–0.805)
NODDI_ICVF		0.620	0.700 (0.608–0.783)	82.6 (0.612–0.950)	58.2 (0.474–0.685)	63.2 (0.536–0.719)	0.569	0.721 (0.584–0.834)	94.1 (0.713–0.999)	63.2 (0.460–0.782)	50.9 (0.372–0.645)
**DCE-MRI**											
Tumor size	Diameter (mm)	27.400	0.548 (0.453–0.642)	60.9 (0.385–0.803)	53.9 (0.431–0.644)	77.9 (0.685–0.852)	22.900	0.558 (0.418–0.629)	94.1 (0.713–0.999)	31.6 (0.175–0.487)	69.1 (0.550-0.805)
	>4 mm/<4 mm	NA	0.532 (0.436–0.626)	26.1 (0.102–0.484)	80.2 (0.706–0.878)	60.3 (0.599–0.774)	NA	0.545 (0.405–0.680)	35.3 (0.142–0.617)	73.7 (0.569–0.866)	61.8 (0.477-0.743)
TIC		NA	0.503 (0.408–0.598)	47.8 (0.268–0.694)	52.8 (0.420–0.633)	79.8 (0.711–0.865)	NA	0.619 (0.478–0.747)	76.5 (0.501–0.932)	47.4 (0.310–0.642)	69.1 (0.550–0.805)

Values in parentheses represented 95% CIs.

AUC, area under the curve; DWI, diffusion-weighted imaging; DSI, diffusion spectrum imaging; DTI, diffusion tensor imaging; DKI, diffusion kurtosis imaging; MAP, mean apparent propagator; NODDI, neurite orientation dispersion and density imaging; MD, mean diffusivity; RD, radial diffusivity; AK, axial kurtosis; NG, non-Gaussianity; NG_Ax_, axial non-Gaussianity; NG_Rad_, radial non-Gaussianity; MSD, mean squared diffusion; QIV, q-space inverse variance; RTOP, return-to-origin probability; RTAP, return-to-axis probability; ICVF, intracellular volume fraction; DCE-MRI, dynamic contrast-enhanced MRI; TIC, time-signal intensity curve; NA, not available.

**Table 5:** Logistic regression analysis of conventional MRI and quantitative DSI parameters in the derivation cohort.

Variables		Univariable logistic regression		Multivariable logistic regression	
		OR (95% CI)	*p*-Value	OR (95% CI)	*p*-Value
Tumor size	Diameter (mm)	0.991 (0.964–1.019)	0.520	–	–
	>4 mm/<4 mm	0.699 (0.241–2.025)	0.509		
TIC		1.023 (0.409–2.557)	0.961	–	–
DTI_MD (10^−3^ mm^2^/s)		0.432 (0.261–0.715)	0.001	–	–
DTI_RD (10^−3^ mm^2^/s)		0.646 (0.413–1.012)	0.057	–	–
DKI_AK		2.256 (1.301–3.903)	0.004	–	–
MAP_NG		2.531 (1.502–4.265)	<0.001	–	–
MAP_NG_Ax_		2.509 (1.498–4.205)	<0.001	–	–
MAP_NG_Rad_		2.515 (1.468–4.308)	0.001	–	–
MAP_MSD (10^−5^ mm^2^)		0.557 (0.349–0.890)	0.014	–	–
MAP_QIV (10^−10^ mm^5^)		0.523 (0.332–0.824)	0.005	–	–
MAP_RTOP (10^5^ mm^−3^)		2.295 (1.288–4.091)	0.005	–	–
MAP_RTAP (10^3^ mm^−2^)		2.402 (1.379–4.186)	0.002	–	–
NODDI_ICVF		2.573 (1.465–4.520)	0.001	2.573 (1.465–4.520)	0.001

OR, odds ratio; TIC, time-signal intensity curve; DSI, diffusion spectrum imaging; DTI, diffusion tensor imaging; DKI, diffusion kurtosis imaging; MAP, mean apparent propagator; NODDI, neurite orientation dispersion and density imaging; MD, mean diffusivity; RD, radial diffusivity; AK, axial kurtosis; NG, non-Gaussianity; NG_Ax_, axial non-Gaussianity; NG_Rad_, radial non-Gaussianity; MSD, mean squared diffusion; QIV, q-space inverse variance; RTOP, return-to-origin probability; RTAP, return-to-axis probability; ICVF, intracellular volume fraction.

## Discussion

Our study showed that a total of eleven DSI quantitative parameters, including DTI_RD, DTI_MD, DKI_AK, MAP_NG, MAP_NG_Ax_, MAP_NG_Rad_, MAP_MSD, MAP_QIV, MAP_RTOP, MAP_RTAP, and NODDI_ICVF, significantly differed between HER2-negative and HER2-positive breast cancers. These DSI quantitative parameters had moderate predictive capability with AUC values ranging from 0.628 to 0.700 in the derivation cohort and from 0.673 to 0.721 in the validation cohort for discriminating HER2-positive tumors from HER2-negative tumors. By contrast, ADC did not statistically differ between the HER2-positive and HER2-negative groups. These results indicate that multiple quantitative parameters derived from DSI have potential clinical value in identifying HER2 status in breast cancer patients.

HER2 status determines whether to carry out targeting therapies, including the HER2-targeted trastuzumab or pertuzumab monoclonal antibodies for breast cancer patients (2). Previously, diffusion MRI has been used as a non-invasive approach to evaluate the pathologic characteristic of breast cancer (12–15). As the most common diffusion model, DWI is widely applied for differentiation between benign and malignant breast diseases in clinical practice, in which ADC is usually derived to measure the motion rate of water molecules, whereas a study by Roknsharifi et al. indicated that no significant correlation was found between ADC values and HER2 status (12). Nonetheless, several studies demonstrated that ADC value was significantly higher in HER2-positive breast cancer than that in HER2-negative breast cancer (22–24). In this study, the ADC value was lower in the HER2-positive group than in the HER2-negative group, but no statistically significant difference was found. This controversy might be associated with a much higher heterogeneity in HER2-positive breast cancer compared with HER2-negative breast cancer. Indeed, a recent study by Kim et al. also showed that HER2-positive breast cancer had high intratumoral kinetic heterogeneity (25). The higher heterogeneity might affect the measurement of the ADC value of breast cancer.

Unlike ADC value, our study demonstrated that multiple DTI- and DKI-based parameters, including DTI_RD, DTI_MD, and DKI_AK, differed between the two groups. The HER2-positive group had a higher DTI_RD and DTI_MD than the HER2-negative group. DTI_RD and DTI_MD are characterized by revealing the diffusion rate of water molecules and reflecting the integrity of microstructure (14). A meta-analysis showed that the DTI-based diffusion parameter was not significantly different between HER2-negative and positive breast cancers (26). However, the motion of water molecules in heterogeneous breast tissue does not completely obey Gaussian distribution (27), which is the mathematical and physical frame of DTI measurement. In addition, DSI can describe complex fiber tissues with no need for any intricate models. Thus, the DTI_RD and DTI_MD derived from DSI should be more sensitive to the nonrandom direction of water molecules, which is better suitable for detecting water motion in highly heterogeneous HER2-positive breast cancer. In our study, DKI_AK was lower in the HER2-positive group compared with the HER2-negative group, which was consistent with the result of a previous study by You et al. (28). DKI_AK is a kurtosis coefficient that is characterized by providing water diffusion information along the main diffusion direction (29). It has been demonstrated that neovascularization, vascular permeability, and extracellular fluid are increased in HER2-positive breast cancer (30). These histologic properties might result in high DKI_AK. Meng et al. reported that DKI_MK rather than DKI_AK was significantly decreased in HER2-positive breast cancer (15). This difference might be related to the high cellularity in HER2-positive breast cancer that blocks the water molecular diffusion and partly counteracts the effect of enriched blood perfusion on promoting the movement of water molecules.

In our study, MAP-MRI-based parameters were derived from DSI, such as MAP_NG, MAP_NG_Ax_, MAP_NG_Rad_, MAP_RTOP, MAP_RTAP, MAP_MSD, and MAP_QIV. Among them, MAP_MSD and MAP_QIV were higher in the HER2-positive group compared with the HER2-negative group, and MAP_NG, MAP_NG_Ax_, MAP_NG_Rad_, MAP_RTOP, and MAP_RTAP were lower in the HER2-positive group compared with the HER2-negative group. MAP-MRI is rooted in a q-space framework and relates the diffusion signal measured by Fourier transformation to the three-dimensional probability distribution of water molecule displacement (16). MAP_NG, MAP_NG_Ax_, and MAP_NG_Rad_ can be considered alternatives to the kurtosis metrics (31). Both MAP_RTOP and MAP_RTAP are zero-displacement probability metrics affected by the diffusion of water molecules (16). Our study showed that five parameters were lower in the HER2-positive group than that in the HER2-negative group, which might be explained by the higher cellular density and the higher rate of cell proliferation and cellularity in HER2-positive breast cancer (32). In addition, MAP_MSD and MAP_QIV were also found to be higher in the HER2-positive group compared with the HER2-negative group. MAP_MSD is better than DTI_MD in detecting restricted diffusion of water molecules (33). MAP_QIV is a pseudodiffusivity measure that can represent different diffusion components (34). HER2-positive breast cancer is characterized by rich perfusion and high cellular density, which might be related to the lower MSD and QIV. Additionally, NODDI_ICVF was found to be significantly lower in the HER2-positive group compared with the HER2-negative group in our study. NODDI is a biophysical model with low complexity based on *a priori* assumption about cellular compartments and neural processing orientations (7). NODDI_ICVF represents the diffusion of water molecules within the axons and cells in the central nervous system. The decreased NODDI_ICVF is probably related to the high cellularity of HER2-positive breast cancer.

In this study, ROC analyses showed that the AUC values of ADC and DSI quantitative parameters ranged from 0.616 to 0.713 in identifying HER2 status in the derivation cohort. The AUC of NODDI_ICVF was the highest (0.700), which outperformed ADC and the other DTI-, DKI-, MAP-, and NODDI-based diffusion parameters. Similar results were found in the validation cohort, where NODDI_ICVF also reached the highest AUC (0.721). Notably, univariable and multivariable logistic analyses showed that only NODDI_ICVF was an independent predictor of HER2 status but showed a moderate performance. Previously, the tumor size and TIC type were found to be marginally associated with the HER2 status in breast cancer (15, 35). However, in our study, the tumor size and TIC type were not selected as independent predictors. These results suggest that the DSI quantitative parameter, NODDI_ICVF, is superior to the conventional MRI parameters in predicting HER2 status. The clinical value of DSI is worth to be investigated further.

Our study had several limitations. First, the HER2-positive group had a relatively small sample size (20.18%) in the derivation cohort (114 participants). This imbalanced proportion of molecular types of breast cancer might cause a bias, and no hierarchical analysis was performed in our study. However, the incidence of HER2-positive subtype is not high, which compromises 15%–30% of breast cancer clinically. In addition, the minimum sample size of the HER2-positive group was estimated to be 21 participants by using a power of 0.9 and a type I error rate of 0.05 in our study. Thus, the result of our study is worthy for further validation with a large sample size of patients with HER-2 positive breast cancer. Second, the DSI quantitative parameters were measured by a two-dimensional ROI. A three-dimensional volume of interest containing the whole tumor might provide more information about the tumor compared with two-dimensional ROI, which remains to be validated by further study. In addition, the delineation of the volume of interest was laborious and time-consuming. This problem might be overcome by using an auto-marking method such as artificial intelligence. Third, most DSI quantitative parameters showed a moderate performance in predicting HER2 status. NODDI_ICVF achieved the highest AUC values in the derivation and validation cohorts. When combined with DSI quantitative parameters, conventional MRI parameters, including the tumor size and TIC type, were not selected as independent predictors of HER2 status. Thus, these two parameters seemingly cannot increase the predictive performance of NODDI_ICVF. The predictive performance of DSI remains to be improved.

In conclusion, our study demonstrated that DKI_AK, MAP_NG, MAP_NG_Ax_, MAP_NG_Rad_, MAP_RTOP, MAP_RTAP, and NODDI_ICVF were lower in HER2-positive breast cancer than in HER2-negative breast cancer, and DTI_RD, DTI_MD, MAP_MSD, and MAP_QIV were higher in HER2-positive breast cancer than in HER2-negative breast cancer. DSI quantitative parameters outperformed conventional ADC values in discriminating HER2-positive breast cancer from HER2-negative breast cancer. DSI could be helpful for preoperative prediction of HER2 status, but DSI alone may not be sufficient in predicting HER2 status preoperatively in patients with breast cancer.

## Data Availability Statement

The original contributions presented in the study are included in the article/supplementary material. Further inquiries can be directed to the corresponding authors.

## Ethics Statement

The studies involving human participants were reviewed and approved by the Ethics Committee of Sun Yat-Sen Memorial Hospital, Sun Yat-Sen University. The patients/participants provided their written informed consent to participate in this study.

## Author Contributions

XZ and CM: conceptualization and methodology. WJ, XY, and DW: resources, investigation. XZ and JH: Investigation and formal analysis. CM and ZY: software, data curation, and formal analysis. JS: project administration and supervision. All authors read and approved the final manuscript.

## Funding

This work was supported by Guangdong Province Universities and Colleges Pearl River Scholar Funded Scheme (2017), the National Natural Science Foundation of China (82102130), the Key Areas Research and Development Program of Guangdong (2019B020235001), and the Natural Science Foundation of Guangdong Province (2021A1515010385).

## Conflict of Interest

MW and XY were employed by the Department of MR Scientific Marketing, Siemens Healthcare.

The remaining authors declare that the research was conducted in the absence of any commercial or financial relationships that could be construed as a potential conflict of interest.

## Publisher’s Note

All claims expressed in this article are solely those of the authors and do not necessarily represent those of their affiliated organizations, or those of the publisher, the editors and the reviewers. Any product that may be evaluated in this article, or claim that may be made by its manufacturer, is not guaranteed or endorsed by the publisher.
